# The use of fulvestrant before chemotherapy improves survival in hormone-positive breast cancer: a real-life study

**DOI:** 10.55730/1300-0144.5495

**Published:** 2021-08-26

**Authors:** Deniz Can GÜVEN, Hasan Çağrı YILDIRIM, Enes ERUL, Taha Koray ŞAHİN, İbrahim Yahya ÇAKIR, Oktay Halit AKTEPE, Neyran KERTMEN, Ömer DİZDAR, Sercan AKSOY

**Affiliations:** 1 Department of Medical Oncology, Hacettepe University Cancer Institute, Ankara, Turkey; 2 Department of Internal Medicine, Hacettepe University Faculty of Medicine, Ankara, Turkey

**Keywords:** Fulvestrant, hormone-positive, breast cancer, real-life, FALCON study

## Abstract

**Background/aim:**

We aimed to evaluate the efficacy of fulvestrant and its affecting clinical factors, including the optimal sequencing of fulvestrant and chemotherapy in a real-life cohort.

**Methods:**

The data of 256 metastatic hormone-positive breast cancer patients treated with fulvestrant were evaluated. The association of clinical factors with survival was analyzed with Kaplan-Meier and Cox-regression analyses.

**Results:**

The median age of patients was 57 years. More than half of the patients used fulvestrant in later lines and after chemotherapy (75.8%). The median progression-free (PFS) and overall survival (OS) of all cohort were 6.05 ± 0.56 and 29.70 ± 1.61 months, respectively. Primary endocrine resistance (HR: 1.989, 95% CI: 1.430–2.766, <0.001), use of fulvestrant after chemotherapy (HR: 1.849, 95% CI: 1.182–2.891, p = 0.007) and visceral metastases (HR: 1.587, 95% CI: 1.128–2.233, p = 0.008) were associated with decreased OS in multivariate analyses. Sixteen patients were treated with trastuzumab and fulvestrant combination. The overall response rate (p = 0.340), disease control rate (p = 0.076), and OS (p = 0.289) and PFS (p = 0.276) were similar to overall cohort.

**Conclusion:**

In our experience, fulvestrant treatment was associated with comparable OS to clinical trials in a large cohort of patients. Patients treated with fulvestrant before chemotherapy were garnered significantly more benefit.

## 1. Introduction

Breast cancer is a global health problem and the most important cause of cancer mortality in women worldwide [1]. Significant improvements have been made in the last 20 years in metastatic breast cancer with the advent of targeted therapies in HER-2 positive disease and endocrine treatments in hormone-positive disease [2,3]. Fulvestrant and cyclin-dependent kinase (CDK) inhibitors were added to treatment armamentarium in addition to tamoxifen and aromatase inhibitors in metastatic hormone-positive breast cancer in the last decade and are widely used in clinical practice [4].

Fulvestrant is an estrogen receptor antagonist that blocks estrogen receptor dimerization and DNA binding with the presumed elimination of tamoxifen’s agonist effects [5]. The phase III FALCON demonstrated the superiority of fulvestrant over aromatase inhibitors. In this study, aromatase inhibitor and fulvestrant treatments were compared in patients with hormone-positive metastatic breast cancer who had not previously received endocrine therapy. Progression-free survival was 16.6 months in the fulvestrant arm and 13.8 months in the aromatase inhibitor arm, and fulvestrant became one of the standards of care options in the first-line of treatment of hormone-positive metastatic breast cancer [6].

Although fulvestrant has gained an important place in treating metastatic hormone-positive breast cancer and is widely used in clinical practice, real-life data is scarce. In addition, fulvestrant is often used after chemotherapy in daily practice, but data on this practice is also very limited. In a multicenter study from Italy, the data of 490 breast cancer patients treated with fulvestrant were evaluated and reported an overall survival of 26.8 months. However, a high frequency of endocrine sensitivity (80%) and use of fulvestrant as maintenance after chemotherapy in a significant percentage of patients hardened the generalizations of the results, especially to limited-resource settings [7]. From these points, we aimed to evaluate the efficacy of fulvestrant and affecting clinical factors, including the optimal sequencing of fulvestrant and chemotherapy in a real-life setting.

## 2. Methods

### 2.1. Patients

The medical records of metastatic hormone-receptor-positive breast cancer patients treated with fulvestrant between 09.2005 and 01.2020 in Hacettepe University Cancer Center were retrospectively evaluated. All patients treated in the prespecified dates were included in the analyses other than patients treated in the context of clinical trials, patients treated with fulvestrant and other endocrine treatment combinations.

Baseline demographic features (age, sex, menopause status), histologic subtypes, HER-2 expression status, tumor grades, patterns of metastases (visceral, soft tissue, bone), endocrine sensitivity status (primary resistance, secondary resistance, and endocrine sensitive), the use of trastuzumab with fulvestrant and previous lines of treatment before fulvestrant recorded together with the best response under treatment, date, and pattern of progression under fulvestrant, clinical benefit rate [8] and survival data. Endocrine sensitivity was categorized according to the definitions in the 5th ESO-ESMO international consensus guidelines for advanced breast cancer (ABC5) guideline[9], and CBR was defined as the best response of complete response, partial response, or stable disease lasting more than 24 weeks as suggested in the previous clinical trials [6],[10].

### 2.2. Statistical analyses

The descriptive features were expressed with medians, standard errors, frequencies and percentages wherever appropriate. Baseline characteristics of the fulvestrant plus trastuzumab cohort and the remaining patients were compared with chi-square and Fischer’s exact test. Survival times were reported with medians and standard errors (se). The overall survival (OS) time was defined as the period from treatment initiation to the last follow-up and/or death, and progression-free survival (PFS) time was defined as the period between treatment initiation to disease progression and/or death. Survival analyses were conducted with Kaplan-Meier analyses and comparisons of survival times were between prognostic subgroups were done using the log-rank test. Multivariate survival analyses were conducted by a backward Cox-regression model, including the statistically significant parameters in the univariate analyses, and hazard ratios were calculated together with 95% confidence intervals (CI). Statistical Package for Social Sciences version 20 program was used in the analyses. P-values below 0.05 were considered statistically significant.

## 3. Results

### 3.1. Baseline characteristics

A total of 256 patients were included in the study. The median age of patients was 56.75 ± 0.71, and almost all patients were female (99.2%). The invasive carcinoma of no special type (NST) was the most common histology (72.9%), followed by the invasive lobular carcinoma (10.8%) and mixed histologies (10.8%). Most of the patients had moderate to poorly differentiated tumors (91.5%). Most of the patients’ tumors were positive for ER+ and PR+ while there were a small number of ER+, PR- and ER-, PR+ cases. A small group of patients (6.25%) had HER-2 positivity defined by a 3+ IHC or FISH positivity for HER-2. More than half of the patients used fulvestrant in the later lines of treatment (50.7%) and after chemotherapy (75.8%). Although only 20.3% of the patients used fulvestrant as the first-line of treatment, the use of fulvestrant as first-line treatment (p < 0.001) and before chemotherapy (p = 0.001) was significantly increased after the publishing of the FALCON study [6]. A significant portion of the patients (87.1%) had some level of endocrine resistance. While the bone-only disease was present in 41.4% of the patients, 52.4% of the patients had visceral involvement (Table 1).

### 3.2. Univariate and multivariate survival analyses

The median follow-up after the start of fulvestrant was 18.18 ± 1.20 months. The overall response rate was 35.9%, and the disease control rate was 53.8%. CBR at the 24th week was achieved in 46.9% of the patients. During the follow-up, 211 of 256 patients had progressed or died. The median PFS and OS of all cohort were 6.05 ± 0.56 and 29.70 ± 1.61 months, respectively. In patients with a partial or complete response to fulvestrant, median OS was more than four years and significantly improved compared to patients without response (49.61 ± 1.94 vs. 24.48 ± 2.51, p < 0.001). While the efficacy of fulvestrant was similar in patients with different ages (≥ vs. <65, p = 0.641), menopausal status (p = 0.507) and histologic subtype (p = 0.500), body mass index (<25 or ≥25, p = 0.614); primary endocrine resistance (p < 0.001), use of fulvestrant in later than the first-line (p = 0.003) or after the chemotherapy (p = 0.001) and presence of visceral metastases (p = 0.001) were associated with a decreased survival with fulvestrant (Figure abc, Table 2). The PFS analyses were consistent with OS analyses other than longer PFS in older patients (≥ vs. <65, p = 0.017).

Among these factors, primary endocrine resistance, the use of fulvestrant after chemotherapy, and visceral metastases remained significant in the multivariate analyses for OS. The primary endocrine resistance and visceral metastases were also related to decreased PFS in the multivariate analyses (Table 3).

### 3.3. Fulvestrant plus trastuzumab cohort

Sixteen patients were treated with a combination of trastuzumab and fulvestrant. The menopausal status, patient age (≥65 vs. <65), tumor grade, metastasis pattern (visceral vs. nonvisceral), endocrine sensitivity, and treatment lines of fulvestrant were similar to the general cohort in patients treated with fulvestrant plus trastuzumab. All patients in this cohort used fulvestrant after chemotherapy. The overall response rate (p = 0.340), disease control rate (p = 0.076), as well as the OS (p = 0.289) and PFS (p = 0.276), were similar to the overall cohort in patients treated with fulvestrant plus trastuzumab combination (Table 4).

## 4. Discussion

In this real-life study, fulvestrant treatment was associated with almost 30 months OS and a significant disease control rate. While the outcomes were improved with the earlier use of fulvestrant, patients with primary endocrine resistance and visceral metastases had significantly worse outcomes.

Fulvestrant is a feasible and cost-effective treatment option in advanced breast cancer [11]. Although a variety of clinical trials in different treatment lines were available, the real-life data is limited. The real-life setting is very different from the clinical trials due to more unfit patients and more patients treated in the later lines due to physician preferences or reimbursement reasons [12]. The most comprehensive real-life data on the fulvestrant use was a recent article from Italy. In the study, 490 patients were prospectively evaluated. The study design differed from our study due to the inclusion of patients using fulvestrant as maintenance after chemotherapy, an indication which is not reimbursed in our country. The median OS was similar to our study (26.8 vs. 29.7 months), while the PFS was longer, possibly due to more patients using fulvestrant in the first-line and patients using fulvestrant for maintenance. The line of treatment and presence of liver metastases was related to PFS, and liver metastases were also related to poorer OS in the study [7]. In another recent study from China, the use of fulvestrant in the later lines was related to a decreased response rate and progression-free survival. On the contrary, visceral metastases did not affect the outcomes, although the low number of cases (n = 60) could have confounded the results [13]. Another real-life study on 306 patients treated with fulvestrant in different treatment lines also reported improved PFS in patients without liver metastases and patients receiving fulvestrant in the first-line or before chemotherapy [14]. In our study, the median OS was 48.95 months in patients treated in the first-line. Similarly, an overall survival of more than 40 months was reported in a similar study with the fulvestrant in the first-line [15]. We think that these figures are comparable to the survival times reported in the first-line CDKi combination trials [16] and support “the earlier, the better” notion with fulvestrant and further strengthen its place in the first-line treatment of metastatic breast cancer as an efficient option.

Several other studies tried to detect clinical features of patients benefitting most from the fulvestrant, but many failed to demonstrate specific features [17]. However, a study reported prolonged PFS with lower (<25) BMI in endocrine-resistant patients, although in the endocrine-sensitive cohort, the OS was lower in the BMI<25 group making the generalizability and interpretation of the study results difficult. Earlier treatment with fulvestrant was related to improved outcomes in this study, while HER-2 positive patients had decreased OS contrary to our study [18]. The use of fulvestrant as monotherapy rather than the combination with trastuzumab could be the reason for different results compared to our study. Despite the notion of dual-targeting opportunity in patients with HER-2 and hormone-positive breast cancer, the development of endocrine and anti-HER2 combinations was relatively slow, [19] possibly due to resistance to conventional endocrine treatments in the HER-2 positive breast cancer [20]. Previous trials of aromatase inhibitors with lapatinib or trastuzumab consistently showed improved PFS and response rates, although the lack of overall survival improvements signified the inevitable endocrine resistance in this patient group [21–24]. We think that fulvestrant could be a better partner to anti-HER2 agents in these combinations due to increased activity and a different mechanism of action. In a multicenter study on the 102 HER-2 positive patients, fulvestrant demonstrated more than 40% clinical benefit rate and retained activity even in the heavily pretreated patients. Only 5 of the patients used concurrent trastuzumab in this study. Interestingly, all five patients experienced a stable disease lasting six or more months, which was very promising [25]. Unfortunately, this combination did not progress in the clinical trials after a phase II study closed due to low accrual (NCT00138125). The interest in the endocrine and anti-HER2 combinations was renewed after the development of CDK 4/6 inhibitors. Improved PFS with the combination of abemaciclib plus fulvestrant plus trastuzumab compared to conventional chemotherapy and trastuzumab combination was very promising in phase II monarchHER study [26]. Further development of this combination is eagerly awaited.

Our study has several limitations. First, most of our patients used fulvestrant in later treatment lines or after the chemotherapy. Our study lacks a comparator arm, so the true benefit of fulvestrant compared to other available treatments is hard to be evaluated from our data. Additionally, the retrospective design and the small number of patients in some subgroups make it hard to reach definitive conclusions in these subgroups, although these results could be hypothesis-generating.

## 5. Conclusion

In our experience, fulvestrant treatment was associated with comparable overall survival and disease control rates to clinical trials in a large cohort of patients in a real-life setting. Patients treated with fulvestrant before chemotherapy and in the first-line were garnered significantly more benefit. In addition, we think that fulvestrant plus trastuzumab could be a chemo-free option in HER-2 positive patients with comparable outcomes to HER-2 negative patients treated with fulvestrant.

## Figures and Tables

**Figure:**
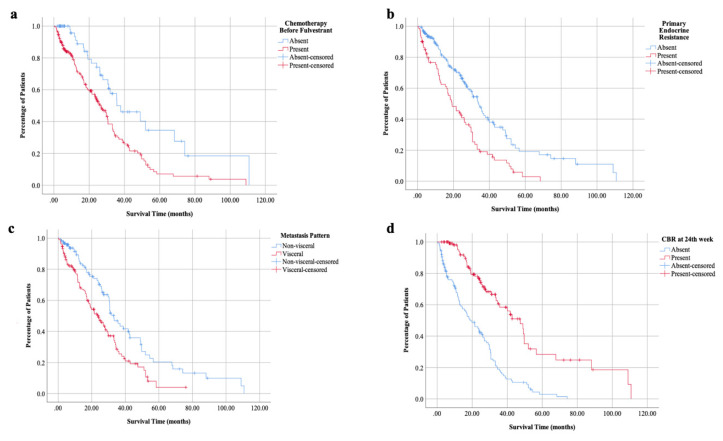
Overall survival according to chemotherapy before fulvestrant (a), primary endocrine resistance (b), metastasis pattern (c) and clinical benefit rate at the 24^th^ week (d).

**Table 1:** Baseline characteristics of patients.

		Count, n	Percentage, %
Sex	Female	254	99.2%
	Male	2	0.8%
Menopausal Status	Premenopausal	115	45.2%
	Perimenopausal	11	4.3%
	Postmenopausal	128	50.5%
Age	<65	198	77.3%
	≥65	58	22.7%
Histologic Type	NST	175	72.9%
	ILC	26	10.8%
	Mixed NST plus ILC	26	10.8%
	Other	13	5.4%
Grade	1	17	8.5%
	2	111	55.2%
	3	73	36.3%
ER positivity	Positive	228	96.6%
	Negative	8	3.4%
PR positivity	Positive	199	86.1%
	Negative	32	13.9%
Endocrine Sensitivity	Endocrine sensitive	33	12.9%
	Primary endocrine resistant	81	31.6%
	Secondary endocrine resistant	142	55.5%
Line of Fulvestrant	1	52	20.3%
	2	75	29.3%
	3	46	18.0%
	4	44	17.2%
	5 or over	39	15.2%
Chemotherapy before Fulvestrant	Absent	62	24.2%
	Present	194	75.8%
Fulvestrant plus trastuzumab	Absent	240	93.7%
	Present	16	6.3%
Metastasis sites	Bone	106	41.4%
	Visceral	55	21.5%
	Bone plus visceral	79	30.9%
	Soft tissue	16	6.3%
Metastasis pattern	Nonvisceral	122	47.7%
	Visceral	134	52.3%
Timing according to FALCON study	Before FALCON study	138	53.9%
	After FALCON study	118	46.1%

* NST: invasive carcinoma of no special type, ILC: invasive lobular carcinoma, ER: estrogen receptor, PR: progesterone receptor

**Table 2:** Median progression-free and overall survival times in univariate analyses.

		PFS (months ± se)	OS (months ± se)
Age	<65	5.52 ± 0.43	29.70 ± 1.55
	≥65	9.53 ± 1.97	31.01 ± 6.99
Menopausal status	Premenopausal	5.39 ± 0.59	28.16 ± 3.22
	Perimenopausal	7.92 ± 1.63	17.08 ± 7.45
	Postmenopausal	7.13 ± 1.22	30.36 ± 1.77
BMI	<25	5.91 ± 1.02	29.70 ± 2.83
	≥25	6.18 ± 0.94	29.14 ± 2.33
Endocrine sensitivity	Endocrine sensitive	18.69 ± 0.78	33.61 ± 5.39
	Primary resistance	3.75 ± 0.26	19.15 ± 3.18
	Secondary resistance	7.23 ± 1.02	33.35 ± 2.51
Metastasis Pattern	Visceral	5.13 ± 0.57	23.89 ± 3.44
	Nonvisceral	8.58 ± 1.92	33.15 ± 2.52
Fulvestrant line	First-line	11.30 ± 1.99	48.95 ± 8.36
	After first-line	5.39 ± 0.41	26.94 ± 2.12
Chemotherapy before fulvestrant	Present	9.46 ± 1.38	26.84 ± 2.07
	Absent	5.59 ± 0.41	35.69 ± 8.45

* BMI: body mass index

**Table 3:** Multivariate analyses for progression-free and overall survival.

Progression-free survival	HR	95% confidence interval	P-value
Primary endocrine resistance	2.345	1.749–3.145	<0.001
Fulvestrant after the first-line	1.280	0.872–1.880	0.208
Chemotherapy before fulvestrant	1.134	0.740–1.738	0.564
Visceral metastases presence	1.361	1.030–1.799	0.030
Age (≥ or <65)	0.716	0.502–1.021	0.065
**Overall survival**	**HR**	**95% CI**	**P-value**
Primary endocrine resistance	1.989	1.430–2.766	<0.001
Fulvestrant after the first-line	1.435	0.753–2.735	0.273
Chemotherapy before fulvestrant	1.849	1.182–2.891	0.007
Visceral metastases presence	1.587	1.128–2.233	0.008

**Table 4:** Comparison of HER-2 positive patients to other patients.

		Fulvestrant plus herceptin cohort (n = 16)	Other patients (n = 240)	P-value
**Age group**	≥65 years of age	2 (12.5%)	56 (23.3%)	0.537
	<65 years of age	14 (87.5%)	184 (76.7%)	
**Grade**	1	0	17 (9%)	0.201
	2	5 (41.7%)	106 (56%)	
	3	7 (58.3%)	66 (35%)	
**Menopause status**	Premenopausal	107 (45%)	8 (50%)	0.827
	Perimenopausal	10 (4.2%)	1 (6.3%)	
	Postmenopausal	121 (50.8%)	7 (43.7%)	
**Metastasis pattern**	Nonvisceral	6 (37.5%)	116 (48.3%)	0.401
	Visceral	10 (62.5%)	124 (51.7%)	
**Treatment line**	First-line	4 (25%)	48 (20%)	0.747
	Second-line or later	12 (75%)	192 (80%)	
**Endocrine sensitivity**	Endocrine sensitive	4 (25%)	29 (12.1%)	0.051
	Primary endocrine resistance	1 (6.3%)	80 (33.3%)	
	Secondary endocrine resistance	11 (68.7%)	131 (54.6%)	
**Overall response (CR/PR)**	Present	4 (25%)	80 (36.7%)	0.340
	Absent	12 (75%)	138 (63.3%)	
**Disease control**	Present	4 (25%)	114 (52.3%)	0.076
	Absent	12 (75%)	104 (47.7%)	
**Progression-free survival, months**		12.75 ± 2.67	5.95 ± 0.53	0.271
**Overall-survival, months**		28.16 ± 13.93	29.7 ± 1.81	0.290
